# Postoperative Endophthalmitis following Cataract Surgery in Asia

**DOI:** 10.5402/2011/917265

**Published:** 2011-12-25

**Authors:** Jin A Choi, Sung Kun Chung

**Affiliations:** ^1^Department of Ophthalmology and Visual Science, St. Vincent Hospital, College of Medicine, The Catholic University of Korea, Seoul, Republic of Korea; ^2^Department of Ophthalmology and Visual Science, St. Mary's Hospital, College of Medicine, The Catholic University of Korea, Seoul, Republic of Korea

## Abstract

Postoperative endophthalmitis is a rare clinical occurrence. However, it remains one of the most serious complications following cataract surgery because of its poor prognosis. We investigated the epidemiologic trends in postoperative endophthalmitis following cataract surgery, particularly in Asian populations. The incidence of postcataract endophthalmitis was generally consistent with epidemiologic data reported from Caucasian populations. The most frequently occurring causative organism was coagulase-negative *Staphylococci* in most studies of Asian populations. However, *Pseudomonas aeruginosa* and *Nocardia* were found to be the most common microorganisms in several studies. The rates of culture positivity were slightly lower than in Caucasian studies. In the evaluation of risk factors of poor visual outcomes, initial visual acuity and virulence of the causative microorganisms were generally found to be the most important risk factors. A history of pars plana vitrectomy was found to be the major risk factors for developing endophthalmitis in several studies.

## 1. Introduction

Postoperative endophthalmitis is the most devastating complication after intraocular surgery, which is commonly associated with a poor prognosis [1]. Postoperative endophthalmitis can occur following any ocular surgery in which the globe is penetrated. However, 90% of postoperative endophthalmitis occurs following cataract surgery, because cataract surgery is one of the most frequently performed intraocular surgeries in the world [2]. Fortunately, postoperative endophthalmitis after intraocular surgery is a rare clinical occurrence, but it often causes severe visual impairment or even the loss of an eye [3]. For advances in sophisticated technique and technology, cataract surgery in developed nations is regarded as a minor procedure with a short recovery time and good results [4, 5]. With this high level of expectation, postoperative endophthalmitis after cataract surgery is especially damaging, because it can lead to permanent vision loss and loss of the eye in severe cases.

Although there are many studies on the diagnosis and treatment guidelines for postcataract endophthalmitis, most of these studies have been conducted in Western countries [1, 4–14]. Therefore, further research is needed on the variation in postoperative endophthalmitis in other countries. In this paper, we investigated the incidence, causative organism, clinical outcomes, and prognostic factors of acute postoperative endophthalmitis after cataract surgery, especially in the Asian population.

## 2. Incidence of Postoperative Endophthalmitis following Cataract Surgery

The incidence of postcataract surgery endophthalmitis varies, ranging from <0.05% to >0.3% [8–16]. This range in the incidence of infection appears to be consistent across numerous patient populations from all over the world [5]. In a study of ten-year incidence of endophthalmitis rate at Bascom Palmer Eye Institute (1984–1994) [17], the incidence of postcataract surgery endophthalmitis was 0.09%. In a meta-analysis of and Taban et al. [13], the overall incidence rate of postoperative endophthalmitis was 0.128% from 1963 to 2003. However, the incidence of postoperative endophthalmitis has changed over time and has increased to 0.265%/year over the last few decades, which coincides temporally with the development of self-sealing clear corneal incisions. Several retrospective, comparative, case-controlled studies found a significantly higher endophthalmitis rate associated with clear corneal incisions compared to sclera tunnel incisions [18–21]. Recently, Nagaki et al. [22] reported a statistically increased risk with clear corneal incisions (0.29%) compared to sclerocorneal incisions (0.05%).

In the Asian population, in a prospective case series which described the incidence of acute endophthalmitis in Singapore from 1996 to 2001, the authors reported an average annual incidence as 0.076% [15]. The incidence rate was relatively lower compared to previous studies, possibly because the study population included large proportions of extracapsular cataract extraction (ECCE) cases. Lalwani et al. [23] investigated postcataract endophthalmitis from 2002 to 2004 in India and reported the average 2-year annual incidence to be 0.05%. In Korea, Kim et al. reported the average incidence of postoperative endophthalmitis following cataract surgery from 1987 to 1994 as 0.23% [24]. However, in a more recent study [25], where the average incidence rate was retrospectively investigated at a single center from 2000 to 2007, it was reported as 0.359%, which was in accordance with the recent increase in the incidence of postcataract endophthalmitis [13]. 

## 3. Causative Organism

Results in the ESCRS postoperative endophthalmitis study, the Endophthalmitis Vitrectomy Study (EVS) [4], and other studies assessing the causative organism demonstrate that Gram-positive organisms account for 90% or more of pathogens isolated in culture-positive cases of postoperative endophthalmitis following cataract surgery, with coagulase-negative *Staphylococci* (i.e., *Staphylococcus epidermidis*) and *Staphylococcus aureus* representing the leading causes [10, 26–28]. In detail, approximately 70% of patients with positive cultures are infected with coagulase negative microorganisms (mostly *Staphylococcus epidermidis*), 10% with *Staphylococcus aureus*, 9% with *Streptococcus* species, 2% with *Enterococcus*, 3% with other Gram-positive species, and finally, 6% with Gram-negative species [26, 29, 30]. In the EVS study, Gram-positive, coagulase-negative micrococci were found to cause less severe infections compared to more virulent Gram-negative, and other Gram-positive organisms, and more virulent organisms caused signs and symptoms of endophthalmitis to appear earlier [4, 6]. 

Table 1 shows the most commonly occurring microorganisms in culture-positive cases in Asia. Likewise in Caucasians, coagulase-negative *Staphylococci* were also found to be the most commonly found isolates [15, 25, 31–33]. However, there were several studies in which *Pseudomonas* species were the most common causative organism [24, 34] (Table 1). In reports from India [35, 36], *Pseudomonas* species and fungi were the most common organisms associated with postoperative endophthalmitis. Uniquely, from a study in India [37], *Nocardia* was found to be the most commonly occurring of all isolates tested. These differences from Asian reports may reflect geographic variations in the causative organism of postcataract endophthalmitis.

In the EVS study, 69.3% of cases demonstrated confirmed microbiologic growth from intraocular specimens [26]. In a more recent study, the culture-positive rate was 66.4% in 250 cases of endophthalmitis referred to a centre in the Netherlands [38]. In Asian study, the culture positive rate was slightly lower than in the Caucasian studies. The rate of culture positivity was 61.8% in Singapore and 52.6% in the India study. In Korea, the culture-positive rate varied from 33.3% to 75% (Table 1) [24, 25, 31–34, 39, 40]. 

## 4. Clinical Outcomes

In the EVS study [4] in 1995, 86% of subjects had an initial acuity of less than 5/200, whereas 26% were light perception only. Three months later, 41% achieved 20/40, and 69% had better than 20/100 vision. Nine months later, 53% achieved 20/40, and 74% were better than 20/100. The most common reason for the loss of visual acuity in the EVS group was attributed to macular lesion, such as epiretinal membrane, macular edema, pigmentary degeneration, and ischemia [4, 7]. In recent studies investigating 250 cases of postcataract endophthalmitis [38], 51.6% had final visual acuities of 20/40 or more at the last examination. In the study of Lalwani et al. [23] from 1996 to 2005, 49.3% of the patients showed visual acuities of 20/40 or more.

In Asian populations, the proportions of patients who showed final visual acuities of 20/40 or more were 29.41% in India [37] and 50.5% in Singapore [15]. In Korea, the proportion varied from 22.2% to 83.3% (Table 2) [24, 25, 31–34, 39, 40], and the proportion of patients who underwent pars plana vitrectomy (ppV) was somewhat larger (23.5 to 93.9%) compared to the studies in Singapore (29.4%) and India (21.1%) (Table 2). Variable initial visual acuities at the time of diagnosis as well as uneven proportion of ppV may explain these variable results in the final visual acuities in Korean studies [24, 25, 31–34, 39, 40]. 

## 5. Risk Factors

In the EVS study [4], multiple independent risk factors associated with poor visual outcomes were found. The strongest risk factor was light perception only vision. Additional risk factors included old age, diabetes, corneal infiltrate or ring ulcer, compromised porterior capsule, low or high intraocular pressure, afferent pupillary defect, rubeosis, and an absent red reflex [4, 41]. Moreover, infecting organisms played an important role in visual prognosis. They reported that a final visual acuity of 20/100 was achieved with Gram-positive, coagulase-negative micrococci 84% of the time, followed by *S. aureus*, 50%, *Streptococci*, 30%, *Enterococci*, 14%, and Gram-negative organisms, 56% [26]. In accordance with these findings, Lalwani et al. and Pijl et al. also emphasized the treatment outcome after endophthalmitis was found to be highly dependent on the causative organism although the outcomes for Gram-negative bacteria and *S. aureus* were better than previously reported [23, 38]. It is reported that some bacteria, such as *S. epidermidis,* may sterilize spontaneously during the ocular inflammatory response [42]. Also, in culture-negative cases of postoperative endophthalmitis, there may be cases with noninfectious inflammation. These may explain the strong effect of virulence of microorganisms on the outcomes of postoperative endophthalmitis.

In the Asian population, the phacoemulsification technique and intraoperative posterior capsule rupture were associated with a 1.8 times higher risk of acute culture-positive endophthalmitis in Singapore [15]. Interestingly, in India, the ECCE technique was found to be the major risk factor for developing endophthalmitis [37]. 

In Korea, the infecting organism was found to play an important role in the visual prognosis [31, 32, 40]. The final visual acuity was found to be associated with the poor initial visual acuity [25, 31]. The history of ppV was associated with poor visual outcomes in several studies [31, 32, 34]. As a treatment option in endophthalmitis, ppV has potential benefits such as reduction of the infecting organism, toxins, inflammatory materials, and opacities as well as collection of samples for culture and potential improvement of intravitreal antibiotic distribution. The ppV in the treatment of endophthalmitis requires highly sophisticated surgical techniques because of corneal edema, severe anterior chamber reaction, and undilated pupil in the patients, which may limit the positive outcomes in endophthalmitis. The need for more complex equipment and an operating room for ppV may delay treatment compared to clinic-based procedures such as vitreous tap and biopsy, which could worsen the outcome [1, 6, 7]. 

In a more recent study, Kuhn and Gini [43] reported that over 90% of the patients who underwent complete vitrectomy achieved >20/40 of final visual acuity. With modern equipment such as adjustable cutting and flow rates as well as better visualization with panoramic viewing systems, ppV after postoperative endophthalmitis is expected to give better outcomes compared to previous studies.

## 6. Conclusions

The incidences of postcataract endophthalmitis were generally consistent with epidemiologic data reported from Caucasian populations. The most causative organism was coagulase-negative *Staphylococci* in most studies carried out with the Asian population. However, *Pseudomonas aeruginosa* and *Nocardia* species were found to be the most common microorganism in several studies. The rates of culture positivity were slightly lower than in Caucasian studies. In the evaluation of risk factors of poor visual outcomes, initial visual acuity and virulence of causative microorganisms was generally found to be the most important risk factors. Studies have shown that a history of ppV have been found to be the major risk factors for developing endophthalmitis.

Globally, postoperative endophthalmitis remains one of the most serious complications following cataract surgery. Early diagnosis and aggressive treatment with antimicrobial therapy as well as surgical intervention is mandatory for optimal visual outcomes. Currently, EVS studies have been standard in the treatment of postcataract endophthalmitis. However, there are local variations, especially with regards to the causative organisms and risk factors in Asian countries. Therefore, for more appropriate treatment of postoperative endophthalmitis, clinicians need to consider these differences in their practice.

## Figures and Tables

**Table 1:** Causative organisms found in postcataract surgery endophthalmitis in Asia.

Study	Years included	Organism detection rate	Most common isolates	Location
Kim et al. [25]	2000–2007	47.4%	*S. epidermidis* (67%)	Korea
			*S. aureus* (11%)	
			*Enterococcus* (11%)	
			*S. pneumoniae* (11%)	

Jung et al. [31]	2001–2006	50.9%	*S. epidermitis* (28%)	Korea
			*Streptococcus sp* (28%)	
			*Enterobacter sp* (17%)	
			*P. aeruginosa* (17%)	
			Fungus (10%)	

Yi et al. [32]	1993–1996	55.6%	*S. epidermidis* (20%)	Korea
			*S. aureus* (10%)	
			*S. viridence* (10%)	
			*P. aeruginosa* (10%)	
			*Serratoa marcescens* (10%)	
			Fungus (30%)	

Choi et al. [34]	1992–1995	75.0%	*Pseudomonas sp* (50%)	Korea
			*S. epidermidis* (42%)	

Kim et al. [24]	1987–1994	33.3% (aqueous humor) 100% (vitreous tap)	*Pseudomonas sp* (50%) *Serratis marcescens* (33%) *S. pneumoniae* (17%)	Korea

Wong and chee [15]	1996–2001	61.8%	*S. epidermidis* (57%)	Singapore
			*Streptococcus sp* (14%)	
			*S. aureus* (10%)	
			*P. aeruginosa* (5%)	
			Other GNB (14%)	

Lalitha et al. [37]	2002-2003	52.6%	*Nocardia* (60%)	India
			*S. epidermidis* (40%)	

**Table 2:** Visual acuity outcomes after postcataract surgery endophthalmitis in Asia.

Study	Years included	Visual acuity at the time of diagnosis (% of ≥20/200)	ppV%	Visual acuity at final followup (% of ≥20/200)	Visual acuity at final followup (% of ≥20/40)
Kim et al. [25]	2000–2007	34.5%	55.1%	62.1%	34.5%
Jung et al. [31]	2001–2006	21.6%	52.3%	77.0%	49.2%
Yi et al. [32]	1993–2006	0.0%	66.7%	50.0%	27.8%
Choi et al. [34]	1992–2005	0.0%	31.2%	75%	50.0%
Kim et al. [24]	1987–1994	33.3%	33.3%	100.0%	83.3%
Park et al.* [33]	1989–1993	3.1%	93.8%	46.9%	25.0%
Wong and chee. [15]	1996–2001	35.3%	29.4%	64.7%	47.1%
Lalitha et al. [37]	2002-2003	21.1%	21.1%	31.6%	26.3%

*This study includes cases with infectious endophthalmitis including traumatic, endogenous, and posttrabeculectomy cases as well as postcataract endophthalmitis.
